# A transcriptomic analysis of *Chrysanthemum nankingense* provides insights into the basis of low temperature tolerance

**DOI:** 10.1186/1471-2164-15-844

**Published:** 2014-10-03

**Authors:** Liping Ren, Jing Sun, Sumei Chen, Jiaojiao Gao, Bin Dong, Yanan Liu, Xiaolong Xia, Yinjie Wang, Yuan Liao, Nianjun Teng, Weimin Fang, Zhiyong Guan, Fadi Chen, Jiafu Jiang

**Affiliations:** College of Horticulture, Nanjing Agricultural University, Nanjing, 210095 China; Jiangsu Province Engineering Lab for Modern Facility Agriculture Technology & Equipment, No. 1 Weigang, Nanjing, 210095 Jiangsu Province China

**Keywords:** Transcriptome, RNA Sequencing, Low temperature tolerance, Ornamental plant

## Abstract

**Background:**

A major constraint affecting the quality and productivity of chrysanthemum is the unusual period of low temperature occurring during early spring, late autumn, and winter. Yet, there has been no systematic investigation on the genes underlying the response to low temperature in chrysanthemum. Herein, we used RNA-Seq platform to characterize the transcriptomic response to low temperature by comparing different transcriptome of *Chrysanthemum nankingense* plants and subjecting them to a period of sub-zero temperature, with or without a prior low temperature acclimation.

**Results:**

Six separate RNA-Seq libraries were generated from the RNA samples of leaves and stems from six different temperature treatments, including one cold acclimation (CA), two freezing treatments without prior CA, two freezing treatments with prior CA and the control. At least seven million clean reads were obtained from each library. Over 77% of the reads could be mapped to sets of *C. nankingense* unigenes established previously. The differentially transcribed genes (DTGs) were identified as low temperature sensing and signalling genes, transcription factors, functional proteins associated with the abiotic response, and low temperature-responsive genes involved in post-transcriptional regulation. The differential transcription of 15 DTGs was validated using quantitative RT-PCR.

**Conclusions:**

The large number of DTGs identified in this study, confirmed the complexity of the regulatory machinery involved in the processes of low temperature acclimation and low temperature/freezing tolerance.

**Electronic supplementary material:**

The online version of this article (doi:10.1186/1471-2164-15-844) contains supplementary material, which is available to authorized users.

## Background

Chrysanthemum (*Chrysanthemum morifolium*) is a popular ornamental plant worldwide [1, 2]. Chrysanthemum plants are susceptible to damage when exposed to prolonged periods of low temperature; therefore, improving their tolerance to cold stress is perceived as an important breeding goal. The high chromosome number and polyploidy of major ornamental species complicate the genetics and capacity for gene discovery [3]. Hence, *C. nankingense* has been considered as a convenient genomic model due to its simple diploid nature. In addition, it displays better tolerance to low temperature as compared to the ornamental polyploid species [4].

Temperature is a major determinant of the geographical distribution and length of the growing season in most plant species [5, 6]. However, episodes of low temperature during the growing season cause a substantial loss in the yield of many temperate crops. During chrysanthemum production, specifically in China, extreme low temperatures in early spring and winter, unusual freezing temperatures during late cold spring, and sudden frosts in fall often lead to growth arrest and block flower buds or inflorescence, which in turn result in significant economic losses every year [7]. Temperate plant species can acquire the ability to withstand a prolonged period of sub-zero temperature if they are previously exposed to a period of low temperature above 0°C; this phenomenon is known as low temperature acclimation [5, 8, 9]. However, the molecular basis of cold acclimation (CA) and low temperature/freezing tolerance in chrysanthemum has not yet been explored. In the present study, we aim to fish out candidate genes underlying the process of CA and response to low /freezing temperature, which will help to elucidate the molecular basis of the cold response in *C. nankingense*, and then improve the chrysanthemum varieties cold tolerance.

In Arabidopsis thaliana and some of the winter cereals, a variety of physiological, biochemical, and molecular changes are known to occur during the low temperature acclimation process [9]. In these species, the physical state of the plasma membrane has been shown to be an important determinant of the plant's ability to sense changes in the air temperature [10–14]. Membrane rigidification leads to an increase in the cytosolic concentration of the Ca^2+^ ion [15], which is regarded as a major regulator of low temperature responsive factor. Certain Ca^2+^-dependent protein kinases have also been recognized as positive regulators [16]. The mitogen-activated protein kinase (MAPK) cascade participates in the low temperature signalling and low temperature tolerance [17]. Three candidate chilling response genes, encoding MAPKKK (MAPK kinase kinase), CLC-D (chloride channel D) and RLK (receptor-like protein kinase) homologues are all up-regulated following chilling stress in Maize [18]. The CBF low temperature-response pathway is well conserved across a diversity of species and is a significant component of tolerance to sub-zero temperatures [7, 19]. The CBF pathway is positively regulated by the circadian clock components CCA1 and LHY [20], while the INDUCER OF CBF EXPRESSION 1 (ICE1) protein acts upstream of CBF in the low temperature response pathway [21]. A high expression of osmotically responsive gene (HOS) 1 acts as a negative regulator of the low temperature response [21, 22]. Salicylic acid (SA) is also involved in the response to low temperature stress [23, 24]. RNA processing and nucleocytoplasmic transport play crucial roles in plant stress [25].

The RNA-Seq platform has become a key technology for quantifying the transcriptional response in nonmodel organisms or those with genome characteristics extremely difficult to whole-genome sequencing [26, 27]. In tea (*Camellia sinensis*), this approach has enabled the recognition of 1,770 differentially transcribed genes (DTGs) induced during low temperature acclimation [28]. A similar transcriptomic analysis of *Jatropha curcas* identified over 3,000 genes as being up- or down-regulated by low temperature [29]. While in *Anthurium sp*., the method of digital gene expression enabled 39 low temperature-inducible transcription factors (TFs) to be identified [30].

Here, the RNA-Seq platform based on Illumina NGS technology was used to characterize the transcriptomic response to low temperature by comparing the different transcriptome of *C. nankingense* plants subjected to periods of sub-zero temperature with or without a prior low temperature acclimation, with a view to gaining a deeper insight into the molecular basis of this physiological adaptation. In addition, the identified candidate genes will be useful for improving adaptation to low temperature and enhancing productivity and geographical distribution.

## Results

### RNA-Seq libraries and reads mapping

An overview of the RNA-Seq reads derived from the six libraries using Illumina HiSeq™ 2000 platform is presented in Table 1 and Additional file 1: Figure S1. The raw sequence data have been deposited in the NCBI Sequence Read Archive (http://trace.ncbi.nlm.nih.gov/Traces/sra_sub/sub.cgi). The number of clean reads per library ranged from 7.01 to 7.47 million, and the total number of nucleotides sequenced from 343,646,114 to 365,975,267 (Accession No. for library A SRS591717; Accession No. for library B1 591719; Accession No. for library B2 591720; Accession No. for library C1 591721; Accession No. for library C2 591722; Accession No. for library CK 591679). The proportion of clean reads was over 99% in each library (Additional file 1: Figure S1). Overall, from library A to library CK, 7,270,059, 7,052,023, 7,013,186, 7,228,380, 7,299,665, and 7,468,883 clean reads were obtained respectively. Correspondingly, 356,232,891, 345,549,127, 343,646,114, 354,190,620, 357,683,585, and 365,975,267 total base-pairs (Table 1) were generated. The clean reads were mapped onto a reference gene database, which included all known *C. nankingense* unigene sequences. Raw sequence data were deposited in the NCBI Sequence Read Archive database under the accession number SRP041330. The proportion of unambiguously mapped reads per library ranged from 77.13% in library C2 to 81.03% (C1), and the proportion of unique matches from 57.64% (A) to 60.89% (C1). As the number of reads increased, the identification rate of new genes slowed, which indicated the saturation around seven million reads (7,013,186 in library B2, 7,468,883 in CK) (Additional file 2: Figure S2).

**Table 1:** **Summary of mapping result**

Sample ID	Total reads	Total base pairs	Total mapped reads	Perfect match	<=2 bp Mismatch	Unique match	Multi-position match	Total unmapped reads
A	7270059 (100.00%)	356232891 (100.00%)	5627806 (77.41%)	4003637 (55.07%)	1624169 (22.34%)	4190635 (57.64%)	1437171 (19.77%)	1642253 (22.59%)
B1	7052023 (100.00%)	345549127 (100.00%)	5480579 (77.72%)	3893154 (55.21%)	1587425 (22.51%)	4099659 (58.13%)	1380920 (19.58%)	1571444 (22.28%)
B2	7013186 (100.00%)	343646114 (100.00%)	5444098 (77.63%)	3862298 (55.07%)	1581800 (22.55%)	4128240 (58.86%)	1315858 (18.76%)	1569088 (22.37%)
C1	7228380 (100.00%)	354190620 (100.00%)	5857437 (81.03%)	4335313 (59.98%)	1522124 (21.06%)	4401306 (60.89%)	1456131 (20.14%)	1370943 (18.97%)
C2	7299665 (100.00%)	357683585 (100.00%)	5629870 (77.13%)	3989898 (54.66%)	1639972 (22.47%)	4215854 (57.75%)	1414016 (19.37%)	1669795 (22.87%)
CK	7468883 (100.00%)	365975267 (100.00%)	5865590 (78.53%)	4203842 (56.28%)	1661748 (22.25%)	4403996 (58.96%)	1461594 (19.57%)	1603293 (21.47%)

### Quantification of transcripts and identification of differentially transcribed genes (DTGs)

**Figure 1:**
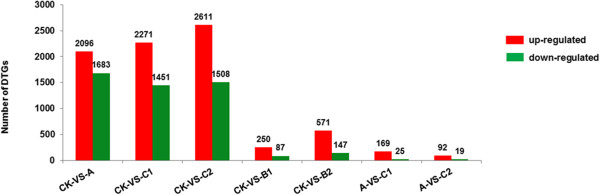
**The numbers of DTGs identified in comparisons between pairs of libraries.**

The quality of the RNA-Seq dataset is assessed by gene coverage, which is the percentage of a gene covered by reads. This value is determined as the ratio of the base number in a gene covered by unique mapping reads to the total bases number of that gene. The distribution of the six libraries was presented in Additional file 3: Figure S3. In addition, transcript abundances for each gene (Additional file 4: Table S1) were calculated according to the method following Mortazavi *et al*. [31]. Moreover, differential transcription was identified through pair-wise comparison between various libraries, by setting a threshold FDR of 0.001 and a│log2 ratio│ of 1 based on the algorithm developed by Audic et al. [32]. From the seven comparisons, including treatment CKA (CK *vs* A), CKB1 (CK *vs* B1), CKB2 (CK *vs* B2), CKC1 (CK *vs* C1), CKC2 (CK *vs* C2), AC1(A *vs* C1), and AC2(A *vs* C2), the results showed that a large number of DTGs were identified (Additional files 5, 6, 7, 8, 9, 10 and 11: Table S2-8). The number of DTGs detected was as follows: treatment CKA, 3,779 (2,096 up- and 1,683 down-regulated); CKB1, 337 (250 and 87); CKB2, 718 (571 and 147); CKC1, 3,722 (2,271 and 1,451); CKC2, 4,119 (2,611 and 1,508); AC1, 194 (169 and 25); and AC2, 111 (92 and 19) (Figure 1). These results indicated that more DTGs were identified in the treatments, which underwent a prior CA (A, C1 and C2), as compared to the treatments which didn’t undergo CA (B1 and B2). In addition, a smaller number of DTGs was found in A *vs* C1 and A *vs* C2 comparisons than in CK *vs* B1 and CK *vs* B2. Moreover, on extending the freezing treatment, fewer DTG were detected in A *vs* C2 comparison than in A *vs* C1. However, contrary results were obtained in case of CK *vs* B1 and CK *vs* B2 comparisons. Based on the assumption that genes with similar expression patterns usually exhibit functional correlation, the consistency of the DTGs was checked by multiple comparisons clustering among the CKA, CKC1 and CKC2; between the CKB1 and CKB2 treatments; and between the comparisons, A *vs* C1 and A *vs* C2. A total of 2,340 DTGs were observed in the first multiple comparison clustering, out of which, only three genes behaved inconsistently (that is, showed up-regulation in one treatment and down-regulation in the other, or vice versa). Of the 2,337 consistent DTGs, 1,410 were up- and 927 were down-regulated (Additional file 12: Table S9). In the second multiple comparison clustering, 188 DTGs (142 up- and 46 down-regulated) were obtained (Additional file 13: Table S10), and all were found to be consistent. In the third comparison clustering between A *vs* C1 and A *vs* C2, 38 DTGs (37 up- and 1 down-regulated) showed consistency (Additional file 14: Table S11).

### GO classification of differentially transcribed genes

**Figure 2:**
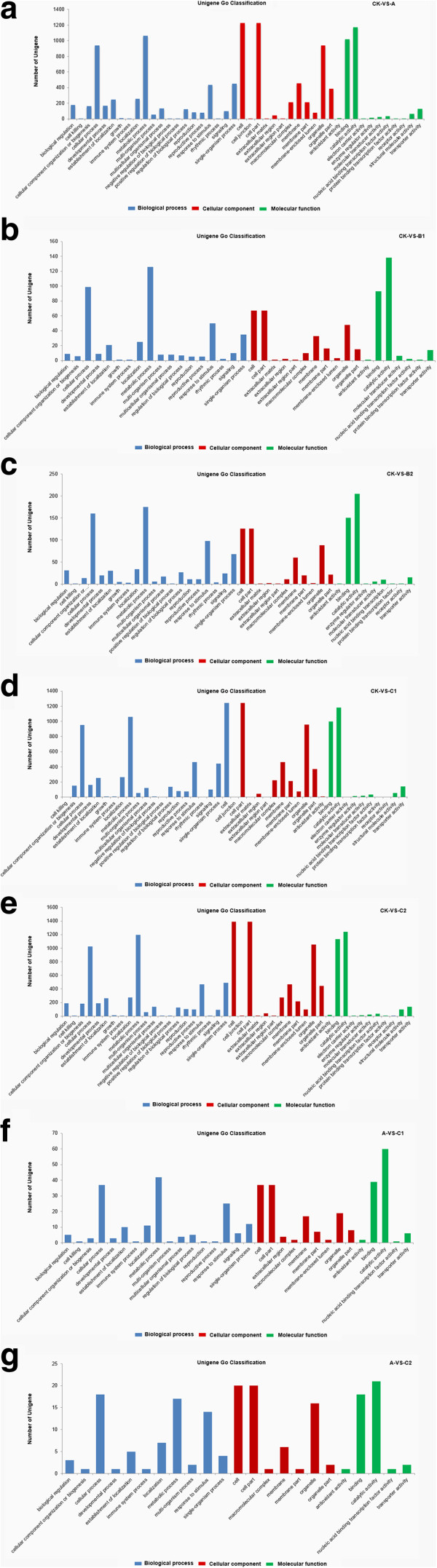
**Gene Ontology (GO) classification of the DTGs identified in each comparison between a pair of libraries.** DTGs were annotated in three categories: biological process, cellular component and molecular function. Y-axis (right) represents the number of DTGs in each category; Y-axis (left) represents the percentage of a specific category of DTGs within that main category. Panels **a, b, c, d, e, f** and **g** (left) represents DTGs in the comparison between library CK (22°C) and A (4°C for one week) (CK-VS-A) (right) , library CK and B1 (-5°C for 1 h) (CK-VS-B1) (right), library CK and B2 (-5°C for 2 h) (CK-VS-B2) (right), library CK and C1 (4°C for one week, followed by -5°C for 1 h) (CK-VS-C1) (right), library CK and C2 (4°C for one week, followed by -5°C for 2 h) (CK-VS-C2) (right), library A and C1 (A-VS-C1) (right), and library A and C2 (A-VS-C2) (right) respectively.

In the treatment CKA, 1,535 of the 3,779 DTGs could be assigned a GO term; the equivalent number for other comparisons were as follows: treatment CKB1, 155/337; CKB2, 246/718; CKC1, 1,522/3722; CKC2, 1,691/4119; A *vs* C1, 60/194; and A *vs* C2, 30/111 (Additional file 15: Table S12). For CK *vs* A, 21 GO classes fell into the categories “biological process”, 12 into “cellular component” and 11 into “molecular function”. The equivalent distribution in CK *vs* B1 was 18, 10, and 7; in CK *vs* B2, 20, 11 and 9; in both, CK *vs* C1 and CK *vs* C2, 21, 12, and 11; in A *vs* C1, 17, 9, and 5; and in A *vs* C2, 11, 7, and 5. The major classes of biological process among the DTGs in CK *vs* A comparison were “metabolic process”, “cellular process”, “single organism process”, “response to stimulus”, “localization”, “establishment localization”, “biological regulation” and “regulation of biological process”; the predominant cellular components were “cell”, “cell part”, “organelle” and “organelle part”; and for molecular function “binding”, “catalytic activity”, “transporter activity”, “nucleic acid binding transcription factor activity” and “antioxidant activity”. Only a few genes belonged to the categories “cell killing”, “electron carrier activity”, “positive regulation of biological process”, “extracellular matrix”, “receptor activity”, “cell junction”, “protein binding transcription factor activity” and “carbon utilization”. The details of GO classification of DTGs in CK *vs* A, and other comparisons are presented in Figure 2. Plant hormone signal transduction pathways (mediated by either auxin or gibberellin) were well represented, particularly those associated with auxin-mediated signalling. Low temperature sensing and signalling genes influenced by Ca^2+^, as well as other protein kinases were also identified. A number of TF families, genes encoding functional proteins and post-translational regulated genes were represented.

### Function annotation of DTGs using the KEGG database

Unigene KEGG annotation was aimed at DTGs from the above comparisons. In the CK *vs* A comparison, 1,972 DTGs were assigned to the KEGG database involved 122 pathways; for CK *vs* B1, 175 DTGs were assigned to 61 pathways; for CK *vs* B2, 302 DTGs were assigned to 75 pathways; for B1 *vs* B2, 100 DTGs were assigned to 46 pathways; for CK *vs* C1, 1,915 DTGs were assigned to 120 pathways; for CK *vs* C2, 2,175 DTGs were assigned to 120 pathways; for A *vs* C1, 90 DTGs were assigned to 46 pathways; and for A *vs* C2, 37 DTGs were assigned to 29 pathways. The details of the KEGG classification of the above comparisons are presented in Additional file 16: Table S13. The major pathways identified were “metabolism”, “biosynthesis of secondary metabolites”, “hormone signal transduction”, “plant-pathogen interaction”, “spliceosome”, “phenylpropanoid biosynthesis”, “endocytosis” and “protein processing in the endoplasm”, “ribosome” and “starch and sucrose metabolism”.

### Genes involved in the response to low temperature

More DTGs were identified in the CK *vs* A, CK vs C1 and CK *vs* C2 comparisons than in the CK *vs* B1 and CK *vs* B2. This observation demonstrated that many genes were involved in low temperature acclimation. The DTGs revealed in the comparisons, A *vs* C1 and A *vs* C2, were found to be involved in response to freezing treatment, when the plants were exposed to prior CA. However, the DTGs obtained from the CK vs B1 and CK vs B2 comparisons participated in the response mechanism, when the plants didn’t undergo prior CA. Some of all the DTGs belonged to the group of genes involved in cold sensing and signal transduction pathways. Ca^2+^ signaling pathway, which is regarded as an important signal sensing and transduction pathway under stresses, includes many members, such as cation/calcium exchangers (CCX), calcium-binding proteins (CBP), calmodulin-like proteins (CML), CBL-interacting protein kinases (CIPK), calcium-dependent protein kinases (CDPK) and calmodulin-binding receptor-like kinases (CBRLK). The CK *vs* A comparison included three *CCX*, two *CBP*, five *CML*, three *CIPK*, and one *CBRLK* genes; the equivalent order for the CK *vs* C1 comparison was, respectively, three, one, five, five and one; for CK *vs* C2, three, zero, four, four and one; for CK *vs* B1, zero, one, three, zero and zero, along with one sodium/calcium exchanger family protein gene (SCE); for CK *vs* B2, zero, zero, five, two, zero, along with one SCE and one CDPK; for A *vs* C1, zero, one, two, one, zero; and for A *vs* C2, only one CBP was involved. In the comparisons, CK *vs* A, CK *vs* C1, and CK *vs* C2, three CCX (CL8580.Contig_S2_3, Unigene82641_S2_3, and Unigene-21268_S2_3), one CML (Unigene74966_S2_1), and one CDPK (Unigene5836_S2_1) were all found to be up-regulated; in addition, two CMLs (Unigene13572_S2_1 and 3462_S2_3), and one CDPK (CL8813.Contig2_S2_1) show-ed down-regulation. One CML (Unigene76667_S2_3) was found to be up-regulated in both comparisons, CK *vs* B1 and CK *vs* B2. These findings indicated that the genes related to Ca^2+^ signaling pathway played an essential role in the phenomenon of CA and freezing response without a prior CA; the complete details of DTGs involved in Ca^2+^ signalling pathway are presented in Table 2. No DTGs associated with the MAPK cascade was identified in the CK *vs* A comparison. In contrast, one *MAPKKK* and one *MAPK* gene were found to be differentially transcribed in CK *vs* C1, and one *MAPKKK* was represented in CK *vs* C2 as well as in CK *vs* B1. Four *MAPKKK* genes were observed in the CK *vs* B2 comparison, and one *MAPKKK* gene was identified in both, A *vs* C1 and A *vs* C2. The results showed that MAPK cascade, especially *MAPKKK* genes, participated in the plant’s response to freezing treatment, instead of CA. Therefore, all the DTGs involved in MAPK cascade were found to be up-regulated (Table 3). A higher number of genes encoding serine/threonine-protein kinases were differentially transcribed in CK *vs* A, CK *vs* C1 and CK *vs* C2 than in CK *vs* B1 or CK *vs* B2 (Table 4). However, no DTG encoding this specific kinase was identified in the A *vs* C1 or A *vs* C2 comparisons.

**Table 2:** **The differential gene expression of genes involved in Ca** ^**2+**^ **signalling pathway in each comparison**

Comparison	GeneID	log2 ratio	Up-Down-Regulation	P-value	FDR	Gene description
CK *vs* A	CL8580.Contig1_S2_3	12.34	Up	1.38E-17	6.12E-16	cation/calcium exchanger4
	Unigene82641_S2_3	5.43	Up	3.53E-12	1.11E-10	cation/calcium exchanger 4-like
	Unigene21268_S2_3	4.98	Up	9.77E-17	4.15E-15	cation/calcium exchanger 4
	Unigene19284_S2_1	-1.46	Down	5.67E-05	0.000728	calcium-binding protein
	Unigene27711_S2_3	-1.30	Down	4.81E-30	3.78E-28	calcium-binding allergen Ole e 8-like
	Unigene74966_S2_1	1.09	Up	2.72E-08	5.82E-07	calmodulin-like protein 1-like
	Unigene13572_S2_1	-2.40	Down	1.22E-33	1.07E-31	calmodulin-like protein 5-like
	Unigene3462_S2_3	-5.71	Down	3.14E-15	1.22E-13	calcium-binding protein
	Unigene76667_S2_3	-1.46	Down	5.29E-13	1.77E-11	calcium-binding protein CML24-like
	Unigene42277_S2_1	-1.40	Down	1.82E-09	4.44E-08	calcium-binding protein CML45-like
	Unigene86355_S2_3	1.00	Up	3.93E-06	0.000062	calmodulin-binding receptor-like cytoplasmic kinase 3-like
	Unigene5836_S2_1	2.01	Up	1.42E-44	1.69E-42	CBL-interacting serine/threonine-protein kinase
	CL4456.Contig1_S2_3	1.07	Up	2.72E-93	7.05E-91	CBL-interacting serine/threonine-protein kinase 6-like isoform 1
	CL8813.Contig2_S2_1	-2.63	Down	6.68E-23	3.94E-21	CBL-interacting serine/threonine-protein kinase 25-like
CK *vs* C1	CL8580.Contig1_S2_3	12.35	Up	6.77E-18	3.12E-16	cation/calcium exchanger 4-like
	Unigene82641_S2_3	5.32	Up	1.92E-11	5.60E-10	cation/calcium exchanger 4-like
	Unigene21268_S2_3	4.56	Up	2.22E-12	7.00E-11	cation/calcium exchanger 4
	Unigene75028_S2_1	1.30	Up	2.91E-07	5.47E-06	calcium-binding protein PBP1-like
	Unigene74966_S2_1	1.37	Up	1.43E-13	4.94E-12	calmodulin-like protein 1-like
	Unigene86214_S2_3	1.20	Up	3.58E-17	1.58E-15	calmodulin-like protein 5-like isoform 1
	Unigene13572_S2_1	-1.44	Down	3.78E-18	1.77E-16	calmodulin-like protein 5-like
	Unigene27042_S2_3	1.07	Up	1.59E-34	1.46E-32	probable calcium-binding protein CML36-like
	Unigene3462_S2_3	-2.20	Down	6.33E-08	1.30E-06	probable calcium-binding protein CML31
	Unigene5836_S2_1	2.50	Up	2.70E-82	6.16E-80	CBL-interacting serine/threonine-protein kinase 6-like isoform 1
	CL2470.Contig1_S2_1	1.64	Up	2.74E-43	3.21E-41	CBL-interacting serine/threonine-protein kinase 6-like isoform 1
	Unigene82472_S2_1	1.52	Up	3.72E-06	5.92E-05	CBL-interacting serine/threonine-protein kinase 11
	Unigene71605_S2_1	1.36	Up	1.24E-54	1.85E-52	CBL-interacting protein kinase 18
	CL8813.Contig2_S2_1	-3.32	Down	2.00E-29	1.56E-27	CBL-interacting serine/threonine-protein kinase 25-like
	Unigene86355_S2_3	1.10	Up	2.01E-07	3.84E-06	calmodulin-binding receptor-like cytoplasmic kinase 3-like
CK *vs* C2	CL8580.Contig1_S2_3	13.00	Up	1.87E-27	1.26E-25	cation/calcium exchanger 4-like
	Unigene82641_S2_3	5.87	Up	1.18E-16	4.68E-15	cation/calcium exchanger 4-like
	Unigene21268_S2_3	5.47	Up	3.91E-24	2.29E-22	cation/calcium exchanger 4
	Unigene13572_S2_1	-2.00	Down	3.91E-27	2.58E-25	calmodulin-like protein 5-like
	Unigene74966_S2_1	1.38	Up	1.46E-13	4.77E-12	calmodulin-like protein 1-like
	Unigene3462_S2_3	-3.72	Down	2.81E-12	8.28E-11	probable calcium-binding protein CML31
	Unigene7543_S2_3	2.17	Up	3.81E-08	0.000000753	probable calcium-binding protein CML10
	CL794.Contig2_S2_1	3.59	Up	0.00000641	0.0000922	CBL-interacting serine/threonine-protein kinase
	Unigene5836_S2_1	2.44	Up	6.21E-76	1.23E-73	CBL-interacting serine/threonine-protein kinase 6-like isoform 1
	CL4456.Contig1_S2_3	1.13	Up	8.1E-107	2.42E-104	CBL-interacting serine/threonine-protein kinase 6-like isoform 1
	CL8813.Contig2_S2_1	-3.00	Down	4.19E-26	2.67E-24	CBL-interacting serine/threonine-protein kinase 25-like
	Unigene86355_S2_3	1.10	Up	0.000000279	0.00000491	calmodulin-binding receptor-like cytoplasmic kinase 3-like
CK *vs* B1	Unigene35781_S2_1	1.13	Up	3.2E-09	0.000000491	sodium/calcium exchanger family protein
	Unigene27711_S2_3	1.26	Up	4.74E-66	1.16E-62	calcium-binding allergen Ole e 8-like
	Unigene76667_S2_3	1.18	Up	4.71E-20	2E-17	calcium-binding protein CML24-like
	Unigene26965_S2_3	1.06	Up	1.16E-10	2.16E-08	probable calcium-binding protein CML48
	Unigene5836_S2_1	1.05	Up	8.75E-10	0.000000144	CBL-interacting serine/threonine-protein kinase 6-like isoform 1
CK *vs* B2	Unigene35781_S2_1	1.15	Up	1.62E-09	0.000000175	sodium/calcium exchanger family protein
	Unigene86214_S2_3	2.02	Up	1.08E-58	1.17E-55	calmodulin-like protein 5-like isoform 1
	Unigene13572_S2_1	1.21	Up	4.51E-30	1.89E-27	calmodulin-like protein 5-like
	Unigene76667_S2_3	1.03	Up	4.6E-15	8.4E-13	calcium-binding protein CML24-like
	Unigene27711_S2_3	1.66	Up	8.69E-131	2.78E-127	calcium-binding allergen Ole e 8-like
	Unigene19284_S2_1	1.22	Up	0.000000074	0.00000616	calcium-binding protein CAST-like
	CL4878.Contig1_S2_1	1.05	Up	2.57E-19	6.24E-17	calcium-dependent protein kinase 9-like
	Unigene5836_S2_1	2.08	Up	1.39E-48	1.16E-45	CBL-interacting serine/threonine-protein kinase 6-like isoform 1
	Unigene82472_S2_1	1.48	Up	0.000011	0.000600274	CBL-interacting serine/threonine-protein kinase 11
A *vs* C1	Unigene27711_S2_3	1.70	Up	1.48E-57	3.91E-54	calcium-binding allergen Ole e 8-like
	Unigene42277_S2_1	1.19	Up	0.000000676	0.0000976	calcium-binding protein CML45-like
	Unigene86214_S2_3	1.19	Up	1.4E-16	7E-14	calmodulin-like protein 5-like isoform 1
	CL2470.Contig1_S2_1	1.39	Up	2.71E-33	3.4E-30	CBL-interacting serine/threonine-protein kinase 6-like isoform 1
A *vs* C2	Unigene27711_S2_3	1.45	Up	2.38E-38	5.5E-35	calcium-binding allergen Ole e 8-like

**Table 3:** **The differential gene expression of MAPK cascades genes in each comparison**

Comparison	GeneID	log2 Ratio	Up-down-regulation	P-value	FDR	Gene description
CK *vs* C1	Unigene36211_S2_1	1.90	Up	1.3E-10	3.55E-09	mitogen-activated protein kinase kinase kinase 3-like
	Unigene36598_S2_1	1.04	Up	0.00000542	8.37E-05	mitogen-activated protein kinase 16
CK *vs* C2	Unigene36211_S2_1	1.93	Up	7.40E-11	1.94E-09	mitogen-activated protein kinase kinase kinase 3-like
CK *vs* B1	Unigene36211_S2_1	1.69	Up	5.43E-08	6.92E-06	mitogen-activated protein kinase kinase kinase 3-like
CK *vs* B2	Unigene34028_S2_3	4.90	Up	2.29E-08	2.06E-06	mitogen-activated protein kinase kinase kinase A-like
	Unigene36211_S2_1	2.24	Up	1.32E-15	2.52E-13	mitogen-activated protein kinase kinase kinase 3-like
	Unigene8656_S2_1	2.12	Up	3.38E-07	2.51E-05	mitogen-activated protein kinase kinase kinase ANP1-like
	Unigene1412_S2_1	1.44	Up	3.32E-09	3.43E-07	mitogen-activated protein kinase kinase kinase ANP1-like
A vs C1	Unigene36211_S2_1	2.01	Up	5E-11	1.47E-08	mitogen-activated protein kinase kinase kinase 3-like
A vs C2	Unigene36211_S2_1	2.03	Up	2.84E-11	1.19E-08	mitogen-activated protein kinase kinase kinase 3-like

**Table 4:** **The differential gene expression of Serine/threonine-protein kinase genes in each comparison**

Comparison	GeneID	log2 ratio	Up-down- regulation	P-value	FDR	Gene description
CK *vs* A	Unigene48078_S2_3	4.56	Up	7.02E-79	1.54E-76	Serine/threonine-protein kinase Nek8
	CL6510.Contig1_S2_3	3.24	Up	1.54E-11	4.57E-10	serine/threonine-protein kinase SRK2I-like
	Unigene24393_S2_1	3.24	Up	8.16E-10	2.05E-08	probable serine/threonine-protein kinase WNK11-like
	Unigene84010_S2_3	2.88	Up	0.00000574	0.0000881	G-type lectin S-receptor-like serine/threonine-protein kinase RLK1-like
	Unigene6145_S2_3	1.86	Up	0.00000809	0.000120748	serine/threonine-protein kinase SRK2B-like
	Unigene82172_S2_1	1.67	Up	5.09E-12	1.58E-10	LRR receptor-like serine/threonine-protein kinase FLS2-like
	CL5751.Contig4_S2_3	1.46	Up	4.35E-29	3.28E-27	serine/threonine-protein kinase GRIK2-like
	Unigene77363_S2_3	1.08	Up	2.51E-14	9.25E-13	receptor-like serine/threonine-protein kinase ALE2-like
	CL2322.Contig2_S2_3	1.00	Up	2.16E-12	6.91E-11	serine/threonine-protein kinase HT1-like
	Unigene51530_S2_3	-2.51	Down	7.52E-08	0.00000153	LRR receptor-like serine/threonine-protein kinase At4g37250-like
	CL2131.Contig1_S2_1	-2.44	Down	1.66E-57	2.62E-55	G-type lectin S-receptor-like serine/threonine-protein kinase RLK1-like
	Unigene74903_S2_1	-2.21	Down	1.68E-14	6.25E-13	LRR receptor-like serine/threonine-protein kinase At4g37250-like
	CL102.Contig1_S2_1	-2.04	Down	0.0000201	0.000280649	serine/threonine-protein kinase At5g41260
	Unigene86891_S2_3	-1.76	Down	0.000000168	0.00000325	LRR receptor-like serine/threonine-protein kinase At1g53430
	Unigene62396_S2_1	-1.75	Down	1.86E-17	8.16E-16	LRR receptor-like serine/threonine-protein kinase At1g07650-like
	Unigene5608_S2_1	-1.71	Down	2.73E-22	1.55E-20	LRR receptor-like serine/threonine-protein kinase At1g56130-like isoform 1
	Unigene82743_S2_1	-1.51	Down	0.000000303	0.00000565	LRR receptor-like serine/threonine-protein kinase At4g37250-like
	CL6249.Contig1_S2_1	-1.15	Down	1.28E-10	3.51E-09	receptor-like serine/threonine-protein kinase At2g45590-like
	Unigene599_S2_1	1.94	Up	5.48E-16	1.08E-13	LRR receptor-like serine/threonine-protein kinase At3g47570-like
	CL528.Contig2_S2_1	1.85	Up	0.0000101	0.000555903	G-type lectin S-receptor-like serine/threonine-protein kinase SD2-5-like
	CL11839.Contig2_S2_3	1.56	Up	0.000000918	0.0000626	G-type lectin S-receptor-like serine/threonine-protein kinase At1g34300-like
	CL11232.Contig1_S2_3	1.46	Up	1.35E-11	1.85E-09	serine/threonine-protein kinase RLCKVII-like
	Unigene7157_S2_3	1.45	Up	7.74E-08	0.00000642	LRR receptor-like serine/threonine-protein kinase GSO1-like
	Unigene82172_S2_1	1.38	Up	6.47E-08	0.00000544	LRR receptor-like serine/threonine-protein kinase FLS2-like
	CL5137.Contig1_S2_1	1.27	Up	2.98E-09	0.00000031	LRR receptor-like serine/threonine-protein kinase GSO1-like
	Unigene35893_S2_1	1.09	Up	1.1E-09	0.000000122	G-type lectin S-receptor-like serine/threonine-protein kinase SD2-5-like
	Unigene13659_S2_3	1.01	Up	0.00000506	0.000296619	inactive leucine-rich repeat receptor-like serine/threonine-protein kinase
	Unigene62396_S2_1	-1.43	Down	2.37E-13	3.75E-11	LRR receptor-like serine/threonine-protein kinase At1g07650-like
CK *vs* C1	Unigene48078_S2_3	4.86	Up	1.6412E-101	4.5942E-99	Serine/threonine-protein kinase Nek8, putative
	Unigene24393_S2_1	3.17	Up	2.28788E-09	5.51317E-08	serine/threonine-protein kinase WNK11-like
	CL6510.Contig1_S2_3	2.74	Up	1.7455E-07	3.36939E-06	serine/threonine-protein kinase SRK2I-like
	Unigene6145_S2_3	2.13	Up	6.15014E-08	1.26115E-06	serine/threonine-protein kinase SRK2B-like
	Unigene77360_S2_3	1.83	Up	5.63286E-05	0.000724311	probable serine/threonine-protein kinase At1g54610-like
	CL8653.Contig4_S2_3	1.72	Up	2.68074E-05	0.000365143	serine/threonine-protein kinase HT1-like
	CL812.Contig8_S2_3	1.65	Up	6.44362E-05	0.000817344	serine/threonine-protein kinase SAPK3
	CL2322.Contig2_S2_3	1.33	Up	1.25092E-23	7.73232E-22	serine/threonine-protein kinase HT1-like
	CL5751.Contig4_S2_3	1.33	Up	5.47232E-24	3.44379E-22	serine/threonine-protein kinase GRIK2-like
	CL3545.Contig5_S2_3	1.19	Up	2.89914E-31	2.39887E-29	serine/threonine-protein kinase At5g41260
	Unigene82172_S2_1	1.09	Up	0.000041836	0.000548576	LRR receptor-like serine/threonine-protein kinase FLS2-like
	Unigene42172_S2_3	1.02	Up	2.83884E-23	1.71985E-21	serine/threonine-protein kinase AtPK2/AtPK19-like
	Unigene85255_S2_3	1.01	Up	1.19271E-05	0.000173177	receptor-like serine/threonine-protein kinase At2g45590-like
	Unigene51530_S2_3	-3.58	Down	7.18962E-11	1.99504E-09	LRR receptor-like serine/threonine-protein kinase At4g37250-like
	Unigene74903_S2_1	-2.56	Down	1.88547E-17	8.4618E-16	LRR receptor-like serine/threonine-protein kinase At4g37250-like
	CL102.Contig1_S2_1	-2.28	Down	3.3508E-06	5.3771E-05	serine/threonine-protein kinase At5g41260
	Unigene74228_S2_1	-2.12	Down	2.61668E-63	4.61793E-61	serine/threonine-protein kinase cx32, putative
	CL2131.Contig1_S2_1	-1.93	Down	1.20746E-44	1.451E-42	G-type lectin S-receptor-like serine/threonine-protein kinase RLK1-like
	Unigene86891_S2_3	-1.90	Down	2.01214E-08	4.3637E-07	LRR receptor-like serine/threonine-protein kinase At1g53430
	Unigene62396_S2_1	-1.74	Down	7.77284E-18	3.56502E-16	LRR receptor-like serine/threonine-protein kinase At1g07650-like
	CL6249.Contig1_S2_1	-1.45	Down	8.25782E-15	3.1245E-13	receptor-like serine/threonine-protein kinase At2g45590-like
	Unigene5608_S2_1	-1.39	Down	2.98124E-17	1.32493E-15	LRR receptor-like serine/threonine-protein kinase At1g56130-like isoform 1
	Unigene28326_S2_1	-1.11	Down	1.13529E-22	6.61992E-21	receptor-like serine/threonine-protein kinase ALE2-like
CK *vs* C2	CL10252.Contig2_S2_1	10.43	Up	0.0000214	0.000279936	serine/threonine-protein kinase HT1-like
	Unigene48078_S2_3	4.65	Up	1.32E-84	2.96E-82	Serine/threonine-protein kinase Nek8, putative
	CL6510.Contig1_S2_3	3.48	Up	3.57E-14	1.21E-12	serine/threonine-protein kinase SRK2I-like
	Unigene24393_S2_1	2.79	Up	0.00000126	0.0000204	probable serine/threonine-protein kinase WNK11-like
	Unigene6145_S2_3	2.32	Up	1.64E-09	3.76E-08	serine/threonine-protein kinase SRK2B-like
	CL8653.Contig4_S2_3	2.21	Up	4.68E-09	0.000000102	serine/threonine-protein kinase HT1-like
	Unigene82172_S2_1	1.81	Up	1.26E-14	4.39E-13	LRR receptor-like serine/threonine-protein kinase FLS2-like
	CL5751.Contig4_S2_3	1.42	Up	2.22E-27	1.48E-25	serine/threonine-protein kinase GRIK2-like
	Unigene98225_S2_3	1.32	Up	0.0000032	0.0000484	probable LRR receptor-like serine/threonine-protein kinase At2g24230-like
	CL1564.Contig1_S2_1	1.15	Up	1.15E-08	0.000000243	serine/threonine-protein kinase AtPK2/AtPK19-like
	CL3545.Contig5_S2_3	1.01	Up	3.54E-21	1.8E-19	probable serine/threonine-protein kinase At5g41260
	CL2492.Contig1_S2_1	-10.17	Down	0.0000845	0.000986827	G-type lectin S-receptor-like serine/threonine-protein kinase At4g27290-like
	Unigene51530_S2_3	-2.71	Down	1.86E-08	0.000000383	probable LRR receptor-like serine/threonine-protein kinase At4g37250-like
	Unigene74228_S2_1	-2.58	Down	1.18E-77	2.4E-75	serine/threonine-protein kinase cx32, putative
	CL2131.Contig1_S2_1	-2.20	Down	3.39E-51	4.56E-49	G-type lectin S-receptor-like serine/threonine-protein kinase RLK1-like
	Unigene74499_S2_1	-2.07	Down	0.0000471	0.000577174	probable leucine-rich repeat receptor-like serine/threonine-protein kinase At5g15730
	CL102.Contig1_S2_1	-2.05	Down	0.0000183	0.000241927	probable serine/threonine-protein kinase At5g41260
	Unigene62396_S2_1	-2.02	Down	4.95E-21	2.49E-19	LRR receptor-like serine/threonine-protein kinase At1g07650-like
	Unigene74903_S2_1	-1.89	Down	4.06E-12	1.18E-10	LRR receptor-like serine/threonine-protein kinase At4g37250-like
	Unigene5608_S2_1	-1.76	Down	3.3E-23	1.86E-21	LRR receptor-like serine/threonine-protein kinase At1g56130-like isoform 1
	Unigene86891_S2_3	-1.33	Down	0.0000185	0.000245014	probable LRR receptor-like serine/threonine-protein kinase At1g53430
	Unigene13930_S2_1	-1.22	Down	1.29E-13	4.23E-12	LRR receptor-like serine/threonine-protein kinase GSO1

In the present study, members of various low tem-perature-responsive transcription factor (TF) families were identified; and 43, 44 and 46 such genes were found to be differentially transcribed in the comparisons, CK *vs* A, CK *vs* C1 and CK *vs* C2, respectively. The major TF families presented were AP2/ERF, bHLH, WRKY and TCP, along with small numbers of MYB, MYC, NAC, DOF, and the trihelix family. The CK *vs* B1 and CK *vs* B2 comparisons showed 8 and 19 TF DTGs, respectively. The number of TF DTGs identified in the CK *vs* B1 or CK *vs* B2 comparison was lesser than the comparisons, CK *vs* A, CK *vs* C1, or CK *vs* C2, which were involved in the process of low temperature acclimation. A larger number of TF DTGs were present in CK *vs* B2 than in the CK *vs* B1 comparison. In addition, five (3 WRKYs, 1 DREB and 1 ERF) and seven TF DTGs (1 DREB, 1 bHLH and 5 ERFs) were found in the A *vs* C1 and A *vs* C2 comparisons, respectively. The identified TF DTGs from the A *vs* C1 and A *vs* C2 comparisons, were involved in response to freezing treatment in plants with prior exposure to CA. The differently transcribed TFs of the CKA treatment, the A *vs* C1, and A *vs* C2 comparisons are presented in Table 5.

**Table 5:** **The differential gene expression of Transcription factors (TFs) in each comparison**

Comparison	GeneID	log2 ratio	Up-down-regulation	P-value	FDR	Gene description
CK *vs* A	CL695.Contig1_S2_3	3.21	Up	1.51E-09	3.71E-08	WRKY transcription factor 4
	Unigene75748_S2_1	-3.51	Down	2.15E-10	5.76E-09	WRKY transcription factor 1
	CL9703.Contig1_S2_1	-3.16	Down	2.7E-14	9.92E-13	WRKY transcription factor 1
	Unigene28584_S2_1	-1.56	Down	7.46E-80	1.66E-77	WRKY transcription factor
	CL4806.Contig1_S2_1	-1.51	Down	2.17E-06	0.0000358	WRKY domain class transcription factor
	Unigene80749_S2_1	-1.37	Down	0.000019	0.000266621	WRKY transcription factor 7-like
	Unigene93511_S2_3	5.77	Up	1.63E-15	6.48E-14	Ethylene-responsive transcription factor
	Unigene79121_S2_1	-10.32	Down	0.0000119	0.000171947	ethylene-responsive transcription factor-like
	CL3891.Contig1_S2_1	-1.35	Down	8.09E-37	7.84E-35	ethylene-responsive transcription factor 5
	Unigene25018_S2_1	-2.85	Down	2E-34	1.81E-32	ethylene-responsive transcription factor 5
	Unigene36230_S2_1	-2.58	Down	3.31E-32	2.78E-30	Ethylene-responsive transcription factor
	Unigene73751_S2_1	-2.38	Down	2.45E-22	1.4E-20	ethylene-responsive transcription factor RAP2-4-like
	Unigene83309_S2_1	3.07	Up	0.0000156	0.000221008	DREBa
	Unigene73473_S2_1	2.33	Up	9.16E-69	1.73E-66	DREB2 transcription factor
	Unigene27271_S2_3	1.39	Up	1.45E-13	5.04E-12	AP2 transcription factor
	Unigene27661_S2_3	1.38	Up	1.74E-57	2.73E-55	AP2 domain class transcription factor
	CL1514.Contig4_S2_1	1.30	Up	9.54E-60	1.55E-57	AP2 domain class transcription factor
	Unigene85419_S2_3	-1.85	Down	1.18E-07	0.00000234	AP2/EREBP transcription factor ERF-2
	Unigene27190_S2_3	1.39	Up	2.77E-72	5.57E-70	TCP family transcription factor TCP4
	Unigene14147_S2_3	1.08	Up	6.58E-41	7.14E-39	TB1-like TCP family transcription factor
	CL1091.Contig3_S2_1	-1.27	Down	3.63E-06	0.0000576	TCP domain class transcription factor
	Unigene97493_S2_3	-2.25	Down	0.0000771	0.000963215	transcription factor bHLH47-like
	CL8515.Contig3_S2_1	-1.04	Down	5.01E-06	0.0000776	transcription factor bHLH13
	Unigene37079_S2_1	2.45	Up	1.16E-25	7.68E-24	transcription factor bHLH128-like
	Unigene28502_S2_1	1.58	Up	3.48E-47	4.44E-45	transcription factor bHLH130-like
	Unigene28413_S2_1	-1.67	Down	8.22E-24	5E-22	transcription factor MYC2-like
	CL2771.Contig2_S2_3	-1.66	Down	8.56E-08	0.00000172	MYC1b transcription factor
	Unigene36464_S2_1	-1.37	Down	1.43E-07	0.0000028	transcription factor MYB44-like
	Unigene56969_S2_3	2.92	Up	2.06E-62	3.51E-60	DOF domain class transcription factor
	Unigene13328_S2_3	1.77	Up	2.7E-105	7.79E-103	trihelix transcription factor GTL2-like
	Unigene84739_S2_3	1.06	Up	2.41E-07	0.00000458	bZIP transcription factor 60-like
	CL12660.Contig2_S2_3	2.17	Up	2.66E-23	1.59E-21	transcription factor GTE1-like
	Unigene43245_S2_1	2.16	Up	0.0000208	0.00028917	transcription factor VIP1-like
	CL9834.Contig2_S2_1	1.69	Up	3.6E-10	9.42E-09	global transcription factor group
	CL3990.Contig3_S2_1	1.25	Up	4.42E-55	6.62E-53	transcription factor BTF3
	Unigene27985_S2_1	1.23	Up	3.7E-11	1.06E-09	transcription factor DIVARICATA-like
	Unigene76924_S2_3	1.18	Up	3E-17	1.31E-15	transcription factor BIM1-like
	Unigene62871_S2_1	-5.49	Down	4.63E-49	6.09E-47	transcription factor HEC1
	CL6640.Contig2_S2_1	-1.70	Down	7.15E-44	8.36E-42	transcription factor, putative
	CL5647.Contig3_S2_3	-1.64	Down	0.0000377	0.000500576	nuclear transcription factor Y
	CL7343.Contig2_S2_1	-1.14	Down	5.47E-09	0.000000126	transcription factor RF2b
A vs C1	CL4444.Contig1_S2_1	10.93	Up	0.0000031	0.000374499	dehydration-responsive element-binding factor 1
	Unigene25410_S2_1	4.68	Up	2.16E-07	0.0000352	WRKY transcription factor 1
	Unigene75748_S2_1	3.54	Up	1.17E-10	3.25E-08	WRKY transcription factor 1
	CL9703.Contig1_S2_1	2.91	Up	2.26E-11	6.85E-09	WRKY transcription factor 1
	Unigene85931_S2_3	1.91	Up	7.27E-29	7.63E-26	ethylene response factor 7
A vs C2	CL4444.Contig1_S2_1	12.92	Up	2.65E-22	2.88E-19	dehydration-responsive element-binding factor 1
	Unigene5394_S2_1	11.27	Up	8.36E-21	8.42E-18	ethylene-responsive transcription factor ERF109-like
	Unigene47799_S2_3	11.06	Up	1.28E-07	0.0000303	ethylene-responsive transcription factor ERF017
	Unigene97493_S2_3	3.83	Up	2.12E-19	1.81E-16	transcription factor bHLH47-like isoform 1
	Unigene13303_S2_3	1.89	Up	3.19E-32	6.01E-29	Ethylene-responsive transcription factor
	Unigene25018_S2_1	1.52	Up	2.98E-07	0.0000653	ethylene-responsive transcription factor 5
	Unigene85931_S2_3	1.27	Up	4.38E-11	0.000000018	ethylene response factor 7

Other relevant classes of protein, which featured as DTGs products, were dehydrin, LEA (late embryogenesis abundant) proteins, heat shock proteins (HSPs). The proteins involved in post-transcriptional regulation, such as ribosomal proteins and a DEAD-box ATP-dependent RNA helicase (RH), were particularly found in the CK *vs* A, CK *vs* C1 and CK *vs* C2 comparisons. Yet, with respect to the above classes of proteins, only one LEA was found in both, A *vs* C1 and A *vs* C2, while two HSPs were identified in the A *vs* C1 comparison. The details of classes of protein of the CKA treatment are presented in Table 6.

**Table 6:** **The differential gene expression of genes encoding LEA protein, HSPs, and RNA helicase in treatment CKA**

Related genes	GeneID	log2 ratio	Up-down-regulation	P-value	FDR	Gene description
*LEA*	CL1591.Contig3_S2_3	11.63	Up	0.00000056	0.0000101	late embryogenesis abundant protein-like protein
	Unigene13900_S2_3	8.57	Up	0	0	late embryogenesis abundant protein 1
	CL3193.Contig2_S2_3	6.39	Up	4.99E-161	2.16E-158	Late embryogenesis abundant protein
	CL11733.Contig3_S2_3	10.83	Up	0	0	LEA1 protein
	CL11733.Contig1_S2_3	8.27	Up	2.69098E-90	6.61827E-88	LEA1 protein
	Unigene28071_S2_1	4.42	Up	0	0	LEA5
	CL3193.Contig2_S2_3	6.39	Up	4.9929E-161	2.158E-158	Late embryogenesis abundant protein Dc3
	Unigene14063_S2_1	-1.16	Down	0.00000526	0.0000813	late embryogenesis abundant protein 3 L-1
*HSPs*	CL337.Contig29_S2_3	2.81	Up	3.39E-11	9.79E-10	putative heat shock protein 90 family protein
	CL1609.Contig2_S2_1	2.29	Up	8.01E-18	3.62E-16	heat shock protein 90
	CL1609.Contig12_S2_1	1.67	Up	1.73E-10	4.67E-09	heat shock protein 90-2
	CL1609.Contig19_S2_1	1.30	Up	2.44E-16	1.02E-14	heat shock protein 90
	CL6923.Contig2_S2_3	1.26	Up	3.84E-71	7.6E-69	heat shock protein, putative
	CL1609.Contig10_S2_1	1.08	Up	5.81E-27	4.02E-25	heat shock protein 90
DEAD-box ATP-dependent RNA helicase	CL4257.Contig4_S2_1	2.38	Up	3.15E-24	1.95E-22	DEAD-box ATP-dependent RNA helicase 56-like
	Unigene6176_S2_3	2.29	Up	6.45E-22	3.62E-20	DEAD-box ATP-dependent RNA helicase 32-like
	CL8809.Contig2_S2_3	2.17	Up	4.76E-07	8.64E-06	DEAD-box ATP-dependent RNA helicase 26-like
	CL4129.Contig1_S2_1	1.82	Up	4.02E-122	1.30E-119	DEAD-box ATP-dependent RNA helicase 31-like
	CL869.Contig2_S2_1	1.45	Up	3.01E-38	3.03E-36	DEAD-box ATP-dependent RNA helicase 28-like
	Unigene233_S2_1	1.21	Up	1.32E-07	2.59E-06	dead box ATP-dependent RNA helicase, putative
	Unigene73562_S2_1	1.21	Up	6.32E-46	7.85E-44	DEAD-box ATP-dependent RNA helicase 47, mitochondrial
	CL6411.Contig2_S2_1	1.12	Up	1.06E-05	0.000155014	dead box ATP-dependent RNA helicase, putative
	Unigene27627_S2_3	1.05	Up	2.71E-07	5.10E-06	DEAD-box ATP-dependent RNA helicase 24-like
	Unigene14283_S2_1	1.04	Up	1.55E-21	8.56E-20	DEAD-box ATP-dependent RNA helicase 21-like
	Unigene97540_S2_3	1.04	Up	4.90E-09	1.13E-07	DEAD-box ATP-dependent RNA helicase 50-like

### Verification of differential transcription using quantitative real time PCR (qPCR)

**Figure 3:**
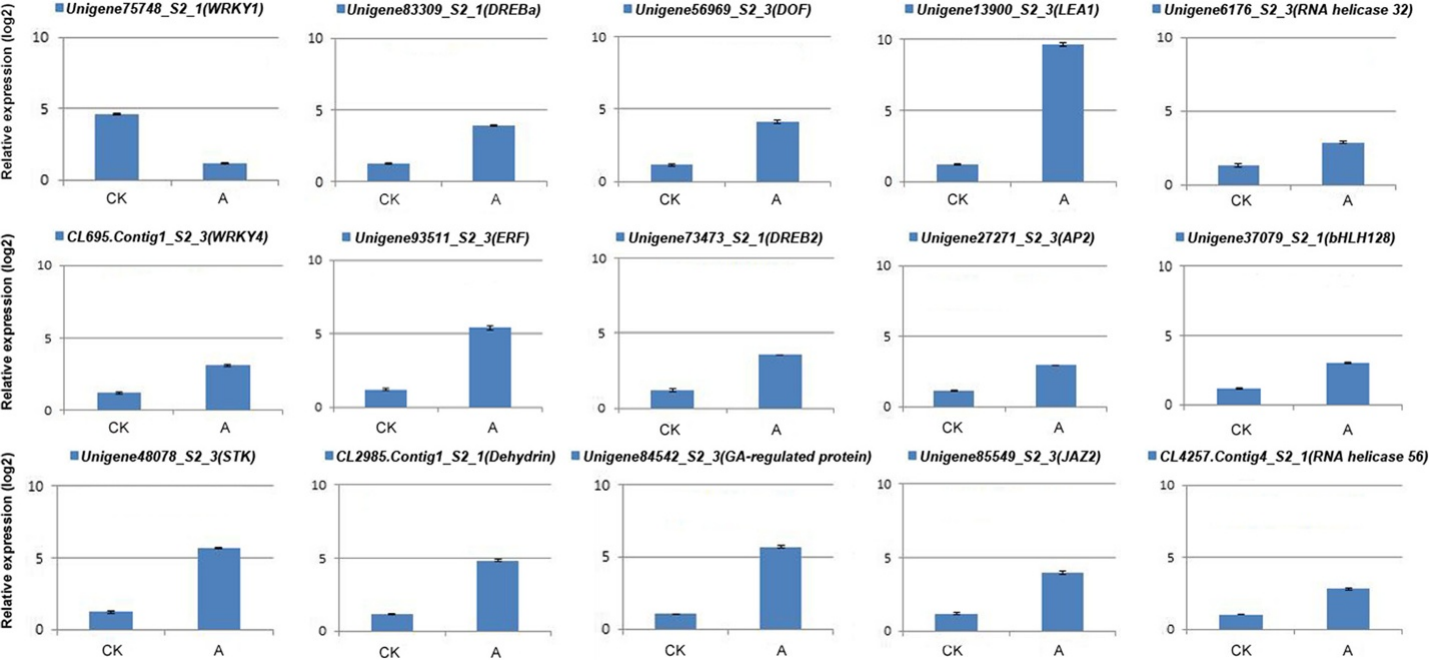
**qPCR validation for 15 DTGs identified by RNA-Seq in the comparison between CK and A.**

To further verify the expression profiles of genes in our Illumina RNA-Seq results, we have performed a selection of 15 DTGs for their key roles in response to low temperature by qRT-PCR, these incorporated genes encoding serine/threonine-protein kinase, LEA protein, dehydrin, a gibberellin-regulated protein, a jasmonate ZIM-domain protein, and a DEAD-box ATP-dependent RH, along with a selection of TFs (WRKY, DREB, AP2, bHLH and DOF). The qPCR outcomes in each case correlated closely with the transcript abundances estimated from the RNA-Seq output (Figure 3).

## Discussion

### Global patterns of transcription in response to low temperature

The information available on the molecular basis of the response of Chrysanthemum to low temperature is still meagre. However, the development of next-generation sequencing technology provides a straightforward method for the identification of genes involved in this process, and we can try to elaborate the molecular mechanism underlying the response to low temperature. Over 77% of the reads in each of the six RNA-Seq libraries corresponded with known transcripts (Table 1), a proportion which was as high as that achieved in a similar study of *Anthurium* [30]. The less than 23% of reads were unmapped probably as a result of unidentified transcripts [33]. Around 4,000 DTGs were identified in each of the CKA, CKC1 and CKC2 treatments (Figure 1). As was also the case for *Camellia sinensis* [28], the majority of DTGs involved up- rather than down-regulation by the stress. In both species, many of the DTGs comprised genes associated with low temperature sensing or signal transduction, low temperature-responsive TFs, stabilization of the plasma membrane and osmosensing-responsiveness.

### Low temperature sensing and signaling genes

Low temperature stress-induced signals are directed to various pathways [8]. Ca^2+^ is well recognized as a messenger in stress signalling [34], and is sensed by proteins of three main classes: CDPKs, CaMs and CBLs [35, 36]. A further important pathway is the mitogen-activated protein kinases (MAPKs) cascade [8], while a number of receptor-like protein kinases (RLKs) are known to be responsible for perceiving changes in the external environment and transducing the appropriate signal [37, 38]. The Arabidopsis and rice genomes harbor, respectively, 50 and 32 CML-encoding genes [39]. In rice, the transcription of the calmodulin-like *OsMSR2* gene is significantly up-regulated by a series of stresses including low temperature in different tissues at different developmental stages, and its heterologous expression in *A. thaliana* has suggested that the gene affects salinity and drought tolerance in an ABA-dependent manner [40]. In *Camellia sinensis* exposed to low temperature, five calmodulin genes, two CDPK genes and one CBL gene were identified to be involved in signal transduction [28]. Here, five Unigenes resembling CML (Unigene74966_S2_1, 13572_S2_1, 3462_S2_3, 76667_S2_3 and 42277_S2_1) were identified as significant DTGs in the CKA treatment, and three of these were also differentially transcribed in both the CKC1 and CKC2 treatments.

CBL-interacting protein kinases (CIPKs), which specifically interact with CBLs, are thought to act as sensors since they lack any enzymatic activity [41]. Three DTGs with homology to CIPK (Unigene5836_S2_1, CL4456.Contig1_S2_3 and CL8813.Contig2_S2_1) were identified in the CKA treatment. Both the CKC1 and CKC2 treatments also featured the same two DTGs (Unigene5836_S2_1 and CL8813.Contig2_S2_1), as well as the other three CIPK homologues (CL2470.Contig1_S2_1, Unigene82472_S2_1, Unigene71605_S2_1) in the former treatment, and two CIPK homologues (CL794.Contig2_S2_1, CL4456.Contig1_S2_3) in the latter. A number of CDPKs have been proven to participate in rapid abiotic stress and immune signaling responses [42]. Transgenic Arabidopsis heterologously expressing the Populus euphratica gene *PeCPK10* show an enhanced level of freezing tolerance, perhaps through the transgene's enhancement of the transcript abundance of the abiotic stress-responsive genes *RD29B* and *COR15A* [43]. Only one *CDPK* homologue (CL4878.Contig1_S2_1) was identified as a DTG in the CKB2 treatment, but no other homologues featured as DTGs in any of the other treatments. However, in A *vs* C1 comparison, with respect to Ca^2+^ signalling pathway, only two *CML* genes (Unigene42277_S2_1 and 86214_S2_3) and one *CIPK* gene (CL2470.Contig1_S2_1) were identified as DTGs. Four *MAPKKK* genes (Unigene34028_S2_3, Unigene36211_S2_1, Unigene8656_S2_1, and Unigene1-412_S2_1) were differently transcribed in the CKB2 treatment. Unigene36211_S2_1 also featured in the CKB1 and CKC2 treatments, while a fifth *MAPK* gene (Unigene-36598_S2_1) was found to transcribe differently in the CKC1 treatment. No one gene of MAPK cascade was identified in the CKA treatment. In addition, many DTGs encoding serine/threonine-protein kinases were found in the CKA, CKC1 and CKC2 treatments; however, no gene was detected in the comparisons, A *vs* C1 and A *vs* C2. These findings provided evidence for the crucial role of Ca^2+^ in the low temperature acclimation process in *C. nankingense*, and further proved that the MAPK pathway and serine/threonine-protein kinases are more strongly involved in the response to freezing.

### Major classes of TF involved in the response to low temperature

Transcriptional regulation of stress-responsive genes is a vital component of the response to both abiotic and biotic stress [44]. Five major TF classes (AP2/ERF, bHLH, WRKY, TCP and MYB) were identified as DTGs in the treatments involved in a process of low temperature acclimation. The AP2/ERF family, which a large group of plant-specific transcription factors, has been sub-divided into AP2, RAV, ERF and DREB TFs [45]. DREBs control the ABA-independent transcription of low temperature responsive genes in *A. thaliana* [10]; the *AtDREB1* sub-family harbors six members [46], of which *DREB1A/CBF3*, *DREB1B/CBF1* and *DREB1C/CBF2* are the ones which respond most rapidly to low temperature. *A. thaliana* plants constitutively expressing any one of these TFs display a heightened tolerance to freezing, drought and salinity [45]. The *DREB2B* TF present in the desert-adapted plant *Eremosparton songoricum* has been shown to enhance the tolerance of both yeast and tobacco against a variety of abiotic stresses. The constitutive expression in tobacco of *EsDREB2B* promotes the accumulation of proline in response to abiotic stress (including low temperature) [47]. Here, the *DREB2* homologue Unigene73473_S2_1 was up-regulated upon exposure to low temperature acclimation. It was speculated that the *DREB2* may be involved in the accumulation of proline in response to low temperature. It is well known Proline accumulates in many plant species in response to environmental stress [48]. The constitutive expression of an *AP2* TF has been shown to improve the tolerance of *A. thaliana* to low temperature, as well as to drought and high temperature [49]. Here, three *AP2*-like genes (Unigene27271_S2_3, Unigene27661_S2_3 and CL1514.Contig4_S2_1) were also all up-regulated by CA. A number of other TFs, belonging to the WRKY, bHLH, TCP, MYB, MYC, Trihelix and b-ZIP families were also among the DTGs identified in treatments involving low temperature acclimation. The class of b-ZIP transcription factors, ABRE binding proteins (AREBs or ABFs), can bind to ABRE and activate ABA-dependent gene expression when plants are exposed to low temperature [10]. Many researches have also indicated that three families of transcription factors: WRKY, bHLH and MYB closely related to plant cold stress [50–52]. Five of six WRKYs, which were detected as DTGs, were down-regulated in CK *vs* A comparison. However, three WRKYs were all up-regulated in A *vs* C1, and no WRKY as DTG was found in A *vs* C2. These findings suggested that WRKY family played essential and different roles in CA and early freezing response. Most ERFs also showed down-regulated in CK *vs* A comparison, while, one ERF was detected up-regulated as DTG in A *vs* C1; on extending the freezing treatment, five ERFs as DTGs were all up-regulated in A *vs* C2. The ERFs performing under different temperature treatment showed its roles in CA and cold tolerance. Few reports to date have indicated that TCP transcription factors involve in the response to cold stress.

### Low temperature responsive genes related to post-transcriptional regulation

Post-transcriptional regulation (pre-mRNA processing, mRNA stabilization and mRNA export from the nucleus) has been implicated in the process of low temperature acclimation [7]. DEAD-box RHs are intimately associated with RNA-mediated processes and are related to various RNA metabolism events, including RNA synthesis to RNA degradation by means of catalyzing the ATP-dependent unwinding of local RNA secondary structures [53]. The transcription of the genes encoding these proteins is known to be regulated by stress in both bacteria and plants [54–57]. In *A. thaliana*, RH25 has been associated with enhanced freezing tolerance, probably through its function as an RNA chaperone [58]. The product of *RCF1*, a low temperature-inducible *RH* gene, is important for low temperature-responsive gene regulation and low temperature tolerance in plants through maintenance of normal pre-mRNA splicing instead of regulating mRNA export like a previously reported DEAD-box RH (LOS4) regulates mRNA export [59]. It has been demonstrated that the functional roles and RNA chaperone activity related to intron splicing in mitochondrial and chloroplast [59–62]. Here, respectively, eleven, nine and twelve genes encoding DEAD-box RHs were up-regulated in the CKA, CKC1 and CKC2 treatments, but none were differentially transcribed in either the CKB1 or the CKB2 treatment; this was taken to imply that DEAD-box RHs are activated during the process of low temperature acclimation. In future, a major task is to clear how RNA chaperones recognize substrate RNAs and how they work with other proteins to regulate post-transcriptional RNA metabolism in response to developmental and environmental condition [53].

### Genes encoding functional proteins

Besides protein factors involved in further regulation of signal transduction and gene expression such as transcription factor and protein kinase that probably function in stress response, various functional proteins featured as products of the DTGs, in particular, LEA protein and dehydrin, LEA proteins are accumulated during the late stage of seed maturation and under moisture deficient conditions, and act to protect higher plants from damage caused by abiotic stress. When the maize gene *ZmLEA3* is expressed in tobacco and yeast, it improves the plant's level of tolerance against both osmotic and oxidative stress [63]. The wheat LEA protein gene *WCI16* expressed heterologously in *A. thaliana* enhances freezing tolerance [64]. Here, seven LEA protein genes were among the DTGs identified in the CKA treatment, five in both the CKC1 and CKC2 treatment, none were present in either the CKB1 or the CKB2 treatment, and only one was identified in both comparisons of A *vs* C1 and A *vs* C2. The implication was that LEA proteins probably enhance low temperature tolerance through their participation in the low temperature acclimation process. Dehydrins constitute a group of plant proteins involved in tolerance to low temperature and drought [65]. The thranscrips of genes encoding three dehydrins in *E. globulus* accumulate strongly in the stem and leaf tissue of acclimated plants, compared to non-acclimated [66]. Here, four genes encoding dehydrins were identified as DTGs in the CKA, CKC1 and CKC2 treatments. But none in either the CKB1 or CKB2 treatments, suggesting that, as with the LEA protein genes, their contribution to low temperature tolerance is expressed during the low temperature acclimation process. HSPs were initially identified from their involvement in the response to high temperatures, but it is now recognized that many of them also respond to low temperature. It is well known HSPs can protect plants against stress by means of reestablishing normal protein conformation and thus cellular homeostasis. Five major HSP families have been conservely defined, based on their molecular weight: HSP100s, HSP90s, HSP70s, HSP60s and small HSPs. Five HSP90-encoding DTGs (all up-regulated) featured in the CKA, CKC1 and CKC2 treatments. HSP90s function as molecular chaperones during signal transduction, cell cycling, the stress response, and in protein folding, degradation and transport [67, 68]. In *A. thaliana*, expression of Hsp90 is developmentally regulated, but it also responds to high and low temperature, as well as salinity [67]. It is speculated that Hsp90 as molecular chaperones play an important role in signal transduction and stress management in *C. nankinginse*. A gene HSP70- encoding DTGs (all down-regulated) featured in the CKA, CKB2, CKC1 and CKC2 treatments, which not found in the CKB1 treatment; and a number of small HSPs, belonging to the 15.7, 22.7 and 23.6 families were also among the DTGs identified in treatments involving low temperature response.

In conclusion, it was clear that a large number of genes were induced when exposed to low temperature. The complex gene networks involved a set of interactions between cold sensing and signaling genes, genes related to post-transcription regulation responding to cold stress, transcription factors and certain functional proteins. In the present study, the DTGs identified as candidate genes in response to low temperature, require further investigation for a complete understanding of the molecular basis of cold response in *C. nankingense*. This information will prove beneficial in molecular breeding programs for excellent chrysanthemum varieties.

## Conclusions

An overview of the many changes to the *C. nankingense* transcriptome induced by exposure to low temperature has been provided. Many of the DTGs identified were involved in predictable classes of gene, encoding, for example, low temperature sensors and signaling molecules, TFs and certain functional proteins associated with the stress response. The large number of DTGs identified confirms the complexity of the regulatory machinery involved in low temperature acclimation and low temperature/freezing tolerance. Establishing which of these many DTGs reflect the primary response to low temperature is necessary before the molecular basis of the response can be fully elaborated; such genes would represent candidates for intervention in the breeding of chrysanthemum varieties endowed with a heightened tolerance to low temperature stress.

## Methods

### Plant material

The accession of *C.nankingense* utilized is conserved by the Chrysanthemum Germplasm Resource Preserving Centre, Nanjing Agricultural University, China. Plants raised from tissue-cultured plantlet were grown on MS medium (16 h photoperiod, 22°C/18°C day/night temperature, 70% relative humidity). Four week old plants were subjected to one of the following temperature treatments: (A) 4°C for one week, (B1) -5°C for 1 h, (B2) -5°C for 2 h, (C1) 4°C for one week, followed by -5°C for 1 h, (C2) 4°C for one week, followed by -5°C for 2 h. CK plants were harvested without any additional treatment. For each treatment, the leaves and stems of three seedlings were sampled, and two samples were harvested. 30 μg of total RNA were pooled in equal amounts from the two biological replicates for subsequent RNA- Seq.

### RNA isolation and cDNA library construction

Six separate libraries were prepared. The samples from the six treatments (A, B1, B2, C1, C2 and CK) were snap-frozen in liquid nitrogen and ground to a fine powder. Total RNA was extracted using a Total RNA Isolation System (Takara, Japan) according to the manufacturer’s instructions. When the quality of the resulting RNA was verified using a 2100 Bioanalyzer RNA Nano chip device (Agilent, Santa Clara, CA, USA), all six extractions delivered an RNA integrity number value of > 8.0, and a 28S:18S ratio >1.5. After checking for the absence of contamination by protein (A260/A280 nm ratios) and reagent contamination (A260/A230 nm ratios) by a Nanodrop ND-430 1000 spectrophotometer, the extractions were selected based on 28S/18S rRNA band intensity (1.5:1 ~ 2:1) and spectroscopic A260/A280 nm readings between 1.8 and 2.2, A260/A230 nm readings greater than 2.0. 10 μg RNA was pooled from each of the three sampled plants. The total RNA preparation was treated with RNase-free DNase I (Takara, Japan) to degrade any possible DNA, and mixed with oligo (dT) coated magnetic beads to concentrate the polyA mRNA.

The mRNA was fragmented into short fragments ~200 nt pieces by incubation in a fragmentation buffer under elevated temperature (The Beijing Genomics Institute). The first strand of cDNA was then synthesized by priming with random hexamer, and the second strand was generated with buffer, dNTPs, RNase H and DNA polymerase I. The double strand cDNA was purified with a QiaQuick PCR extraction kit and resolved with EB buffer for end repair and addition of single nucleotide A. Finally, sequencing adaptors were ligated to the fragments. Following agarose gel electrophore, suitable fragments were selected as templates for PCR.

### RNA-Seq

The library was sequenced using an Illumina HiSeq^™^2000 located at the Beijing Genomics Institute (Shenzhen, China; http://www.genomics.cn/index). The data were deposited in the US National Center for Biotechnology Information (NCBI) Sequence Read Archive (SRA, http://www.ncbi.nlm.nih.gov/Traces/sra; [69] under accession number (SRP041138). Raw data were saved as .fastq files. Data filtering is performed to obtain “clean reads” for further analysis. Clean reads were obtained by removing adaptor sequences, reads in which the percentage of unknown bases (N) was greater than 10% and low quality reads. The clean reads were mapped onto the reference sequences using SOAP (2.21) software [70]. A maximum of two mismatches was allowed in the alignment. The NCBI non-redundant protein (Nr) database (http://www.ncbi.nlm.nih.gov) and the Swiss-Prot protein database (http://www.expasy.ch/sprot) were used for blast search and annotation using an *E*-value cut off of 10^-5^. Functional annotation by gene ontology terms (GO, http://www.geneontology.org) was analyzed using the Blast2GO program [71]. The Kyoto Encyclopedia of Genes and Genomes Pathway (KEGG; http://www.genome.jp/kegg), the major public pathway-related database [72] was also used to predict and classify possible functions. The RPKM reads (clean reads per kilo base per million) method [31] was used to estimate transcript abundance on the base of eliminating the influence of different gene length and sequencing discrepancy. Therefore, the RPKM values can be directly used for comparing the difference of gene expression among samples.

### Identification of differentially expressed genes

To compare the differences in gene expression, the method of an algorithm developed by Audic et al. [32] was used to identify DTGs. The criteria applied were an FDR (false discovery rate) less than 0.01 and an absolute value of log2 ratio of at least 1. Then, the DTGs were subjected to GO and KEGG Ontology (KO) enrichment analysis on the base of a hypergeometric test.

### qPCR validation of differential transcription

Total RNA was isolated from leave and stem of plants subjected to the various treatments described above. Contaminating DNA was removed by treating with RNase-free DNase I and the first cDNA strand was synthesized from 1 μg total RNA using PrimeScript® Reverse Transcriptase (Takara, Dalian, China) and an oligo (dT) primer, according to the manufacturer’s instructions. qPCRs were performed in an Eppendorf Real Time PCR System (Mastercycler®ep realplex, Germany) using a SYBR Premix Ex Taq™ Kit (Takara), according to the manufacturer’s protocol. Gene-specific primers were designed using Primer5 software (sequences given in Table 7). Each 20 μL qPCR contained 5 μL diluted cDNA, 100 nM of each primer, and 10 μl SYBR Green PCR master mix, and was exposed to an initial denaturation (95°C/2 min), followed by 40 cycles of 95°C/15 s, 60°C/15 s, 72°C/15 s. After amplication, all results were screened to verify a single peak melting curve for the specificity of the amplifications. Three biological replicates were performed for each sample. Relative transcript abundance was obtained by including the *C. nankingense EF1α* gene as the reference, and was based on the 2^-ΔΔCT^ method [73].

**Table 7:** **Primers of quantitative reverse transcription-polymerase chain reaction for validation of RNA-Seq data**

GeneID	Primer F (5′-3′)	Primer R (5′-3′)	Blast nr
CL695.Contig1_S2_3	GTGACGAGTTGGTGATGGTG	GTTACCACCTACGAAGGCCA	WRKY transcription factor 4
Unigene75748_S2_1	CGGGTGAAATGCTCTCAAAT	TGCCAAATGGTTCTAAAGGG	WRKY transcription factor 1
Unigene83309_S2_1	ATTTAAACACGCGGATCGAC	CCAGAGTGTGGCTTGGTACA	DREBa
Unigene73473_S2_1	TAAAGGTGGGCCAGAAAATG	ATCATACGCCAGAGCAGCTT	DREB2 transcription factor
Unigene27271_S2_3	ACAACATCCCCTTGGATGAA	GGGTGACAGCATTTGAAGGT	AP2 transcription factor
Unigene93511_S2_3	TGTGCCGCTGTTATCCATTA	CCACACTATCACAGCCCCTT	Ethylene-responsive transcription factor
Unigene37079_S2_1	TCTTCTTTCCCTTTCTGCGA	TGGATCTCCCTCATGACTCC	bHLH128-like
Unigene56969_S2_3	GCATTTGCAGCTGATTCTGA	GCTATCACCGTTGACCCACT	DOF domain class transcription factor
Unigene13900_S2_3	ATCGTGTCGCCGGTATTTAG	GTTGTAGACAAAGCGTCGCA	LEA protein 1
CL2985.Contig1_S2_1	CATCCCCATATTGGTTCCAG	GAACACGAAGCAAGAGGGTC	dehydrin
CL4257.Contig4_S2_1	CTTCTTGCACACTGGTCGAA	GGGGCTTGCTAGGGATAAAG	DEAD-box ATP-dependent RNA helicase 56-like
Unigene6176_S2_3	TGTTTGGCTTGTCAAACTGG	TCCGTGTTATTCCTTTTGCC	DEAD-box ATP-dependent RNA helicase 32-like
Unigene84542_S2_3	CCAGGTTTCGTTTTCGTCAT	GCCTTGAATGCTTTCCACAT	Gibberellin-regulated protein
Unigene85549_S2_3	ACCTCTGTCGGTCCATCAAC	TCGGAACGAGCTCATCTTTT	jasmonate ZIM-domain protein 2
Unigene48078_S2_3	TTTCAGCCGATGGTGATGTA	GTCGTGCCCCACAAGATACT	Serine/threonine-protein kinase

## Electronic supplementary material

Additional file 1: Figure S1: Composition of raw reads in the six RNA libraries. “Clean” reads refers to those remaining after the removal of adaptor sequences, reads in which the proportion of missing bases was >10% and reads in which low quality (≤5) bases represented >50% of the reads. The numbers in parentheses indicate the percentage of each type of read present. (TIFF 616 KB)

Additional file 2: Figure S2: Sequencing saturation analysis in the six libraries (A, B1, B2, C1, C2 and CK). The numbers of new genes detected rose as the read number was increased, but not beyond a threshold around 7,000,000. (TIFF 797 KB)

Additional file 3: Figure S3: Distribution of gene coverage in the six libraries. (TIFF 783 KB)

Additional file 4: Table S1: The transcription level of each unigene derived from the number of relevant reads recovered in the four libraries. The “GeneLength” column gives the length of exon sequence. (XLSX 7 MB)

Additional file 5: Table S2: Genes differentially transcribed in the comparison between libraries CK and A. The criteria applied for assigning significance were: P-value < 0.05, FDR ≤ 0.001, and estimated absolute |log2^Ratio(A/CK)^| ≥1. Genes listed in descending order of absolute |log2^Ratio(A/CK)^|. GeneIDs retrieved from the *Chrysanthemum nankingense* Reference Sequence Database. Annotation of unigene sequences performed using BlastX (E < 10). The “GeneLength” column gives the length of exon sequence. CK- and A- expression: frequency of unigene transcripts in libraries CK and A, respectively. CK- and A-RPKM: reads per kb per million reads for each unigene in libraries CK and A, respectively. Log2^Ratio(A/CK)^: the ratio between the RPKM in CK and the RPKM in A. Up-Down-Regulation (A/CK), P-value and FDR of each gene are also shown. KEGG: annotation according to the KEGG database by BLAST. Blast nr: identification of homologues in GenBank. GO Component, GO Function and Go Process: ontology information of Cellular Components, Molecular Function and Biological Processes of Gene-corresponding GO terms. “-”: no hit. (XLSX 741 KB)

Additional file 6: Table S3: Genes differentially transcribed in the comparison between libraries CK and B1. The criteria applied for assigning significance were: P-value < 0.05, FDR ≤ 0.001, and estimated absolute |log2^Ratio(B1/CK)^| ≥1. Genes listed in descending order of absolute |log2^Ratio(B1/CK)^|. GeneIDs retrieved from *Chrysanthemum nankingense* Reference Sequence Database. Annotation of unigene sequences performed using BlastX (E <10). The “GeneLength” column gives the length of exon sequence. CK- and B1-expression: frequency of unigene transcripts in libraries CK and B1, respectively. CK- and B1-RPKM: reads per kb per million reads for each unigene in libraries CK and B1, respectively. Log2^Ratio(B1/CK)^: the ratio between the RPKM in CK and the RPKM in B1. Up-Down-Regulation (B1/CK), P-value and FDR of each gene are also shown. KEGG: annotation according to the KEGG database by BLAST. Blast nr: identification of homologues in GenBank. GO Component, GO Function and Go Process: ontology information of Cellular Components, Molecular Function and Biological Processes of Gene-corresponding GO terms. “-”: no hit. (XLSX 85 KB)

Additional file 7: Table S4: Genes differentially transcribed in the comparison between libraries CK and B2. The criteria applied for assigning significance were: P-value < 0.05, FDR ≤ 0.001, and estimated absolute |log2^Ratio(B2/CK)^| ≥1. Genes listed in descending order of absolute |log2^Ratio(B2/CK)^|. GeneIDs retrieved from the *Chrysanthemum nankingense* Reference Sequence Database. Annotation of unigene sequences performed using BlastX (E <10). The “GeneLength” column gives the length of exon sequence. CK- and B2-expression: frequency of unigene transcripts in libraries CK and B2, respectively. CK- and B2-RPKM: reads per kb per million reads for each unigene in libraries CK and B2, respectively. Log2^Ratio(B2/CK)^: the ratio between the RPKM in CK and the RPKM in B2. Up-Down-Regulation (B2/CK), P-value and FDR of each gene are also shown. KEGG: annotation according to the KEGG database by BLAST. Blast nr: identification of homologues in GenBank. GO Component, GO Function and Go Process: ontology information of Cellular Components, Molecular Function and Biological Processes of Gene-corresponding GO terms. “-”: no hit. (XLSX 157 KB)

Additional file 8: Table S5: Genes differentially transcribed in the comparison between libraries CK and C1. The criteria applied for assigning significance were: P-value < 0.05, FDR ≤ 0.001, and estimated absolute |log2^Ratio(C1/CK)^| ≥1. Genes listed in descending order of absolute |log2^Ratio(C1/CK)^|. GeneIDs retrieved from the *Chrysanthemum nankingense* Reference Sequence Database. Annotation of unigene sequences performed using BlastX (E <10). The “GeneLength” column gives the length of exon sequence. CK- and C1-expression: frequency of unigene transcripts in libraries CK and C1, respectively. CK- and C1-RPKM: reads per kb per million reads for each unigene in libraries CK and C1, respectively. Log2^Ratio(C1/CK)^: the ratio between the RPKM in CK and the RPKM in C1. Up-Down-Regulation (C1/CK), P-value and FDR of each gene are also shown. KEGG: annotation according to the KEGG database by BLAST. Blast nr: identification of homologues in GenBank. GO Component, GO Function and Go Process: ontology information of Cellular Components, Molecular Function and Biological Processes of Gene-corresponding GO terms. “-”: no hit. (XLSX 787 KB)

Additional file 9: Table S6: Genes differentially transcribed in the comparison between libraries CK and C2. The criteria applied for assigning significance were: P-value < 0.05, FDR ≤ 0.001, and estimated absolute |log2^Ratio(C2/CK)^| ≥1. Genes listed in descending order of absolute |log2^Ratio(C2/CK)^|. GeneIDs retrieved from the *Chrysanthemum nankingense* Reference Sequence Database. Annotation of unigene sequences performed using BlastX (E <10). The “GeneLength” column gives the length of exon sequence. CK- and C2-expression: frequency of unigene transcripts in libraries CK and C2, respectively. CK- and C2-RPKM: reads per kb per million reads for each unigene in libraries CK and C2, respectively. Log2^Ratio(C2/CK)^: the ratio between the RPKM in CK and the RPKM in C2. Up-Down-Regulation (C2/CK), P-value and FDR of each gene are also shown. KEGG: annotation according to the KEGG database by BLAST. Blast nr: identification of homologues in GenBank. GO Component, GO Function and Go Process: ontology information of Cellular Components, Molecular Function and Biological Processes of Gene-corresponding GO terms. “-”: no hit. (XLSX 867 KB)

Additional file 10: Table S7: Genes differentially transcribed in the comparison between libraries A and C1. The criteria applied for assigning significance were: P-value < 0.05, FDR ≤ 0.001, and estimated absolute |log2^Ratio(C1/A)^| ≥1. Genes listed in descending order of absolute |log2^Ratio(C1/A)^|. GeneIDs retrieved from the *Chrysanthemum nankingense* Reference Sequence Database. Annotation of unigene sequences performed using BlastX (E <10). The “GeneLength” column gives the length of exon sequence. A- and C1-expression: frequency of unigene transcripts in libraries A and C1, respectively. A- and C1-RPKM: reads per kb per million reads for each unigene in libraries A and C1, respectively. Log2^Ratio(C1/A)^: the ratio between the RPKM in A and the RPKM in C1. Up-Down-Regulation (C1/A), P-value and FDR of each gene are also shown. KEGG: annotation according to the KEGG database by BLAST. Blast nr: identification of homologues in GenBank. GO Component, GO Function and Go Process: ontology information of Cellular Components, Molecular Function and Biological Processes of Gene-corresponding GO terms. “-”: no hit. (XLSX 53 KB)

Additional file 11: Table S8: Genes differentially transcribed in the comparison between libraries A and C2. The criteria applied for assigning significance were: P-value < 0.05, FDR ≤ 0.001, and estimated absolute |log2^Ratio(C2/A)^| ≥1. Genes listed in descending order of absolute |log2^Ratio(C2/A)^|. GeneIDs retrieved from the *Chrysanthemum nankingense* Reference Sequence Database. Annotation of unigene sequences performed using BlastX (E <10). The “GeneLength” column gives the length of exon sequence. A- and C2-expression: frequency of unigene transcripts in libraries A and C2, respectively. A- and C2-RPKM: reads per kb per million reads for each unigene in libraries A and C2, respectively. Log2^Ratio(C2/A)^: the ratio between the RPKM in A and the RPKM in C2. Up-Down-Regulation (C2/A), P-value and FDR of each gene are also shown. KEGG: annotation according to the KEGG database by BLAST. Blast nr: identification of homologues in GenBank. GO Component, GO Function and Go Process: ontology information of Cellular Components, Molecular Function and Biological Processes of Gene-corresponding GO terms. “-”: no hit. (XLSX 34 KB)

Additional file 12: Table S9: Expression pattern analysis of DTGs following multiple comparisons: CK vs A, CK vs C1, CK vs C2. GeneIDs retrieved from the Chrysanthemum nankingense Reference Sequence Database. Log2^Ratio(A/CK)^: the ratio between the RPKM in CK and the RPKM in A. Log2^Ratio(C1/CK)^: the ratio between the RPKM in CK and the RPKM in C1. Log2^Ratio(C2/CK)^: the ratio between the RPKM in CK and the RPKM in C2. KEGG: annotation according to the KEGG database by BLAST. Blast nr: identification of homologues in GenBank. GO Component, GO Function and Go Process: ontology information of Cellular Components, Molecular Function and Biological Processes of Gene-corresponding GO terms. “-”: no hit. (XLSX 336 KB)

Additional file 13: Table S10: Expression pattern analysis of DTGs following multiple comparisons: CK *vs* B1, CK *vs* B2. GeneIDs retrieved from the *Chrysanthemum nankingense* Reference Sequence Database. Log2^Ratio(B1/CK)^: the ratio between the RPKM in CK and the RPKM in B1. Log2^Ratio(B2/CK)^: the ratio between the RPKM in CK and the RPKM in B2. KEGG: annotation according to the KEGG database by BLAST. Blast nr: identification of homologues in GenBank. GO Component, GO Function and Go Process: ontology information of Cellular Components, Molecular Function and Biological Processes of Gene-corresponding GO terms. “-”: no hit. (XLSX 38 KB)

Additional file 14: Table S11: Expression pattern analysis of DTGs following multiple comparisons: A vs C1, A vs C2. GeneIDs retrieved from the Chrysanthemum nankingense Reference Sequence Database. Log2^Ratio(C1/A)^: the ratio between the RPKM in A and the RPKM in C1. Log2^Ratio(C2/A)^: the ratio between the RPKM in A and the RPKM in C2. KEGG: annotation according to the KEGG database by BLAST. Blast nr: identification of homologues in GenBank. GO Component, GO Function and Go Process: ontology information of Cellular Components, Molecular Function and Biological Processes of Gene-corresponding GO terms. “-”: no hit. (XLSX 17 KB)

Additional file 15: Table S12: GO classification of DTGs in each comparison. (XLSX 194 KB)

Additional file 16: Table S13: Pathway classification of DTGs in each comparison. (XLSX 72 KB)
