# Supplementary figures and images for: A transcriptomic analysis of Chrysanthemum nankingense provides insights into the basis of low temperature tolerance
Source: BMC Genomics. 2014 Oct 3;15(1):844. doi: 10.1186/1471-2164-15-844 (PMC4197275; doi:10.1186/1471-2164-15-844)

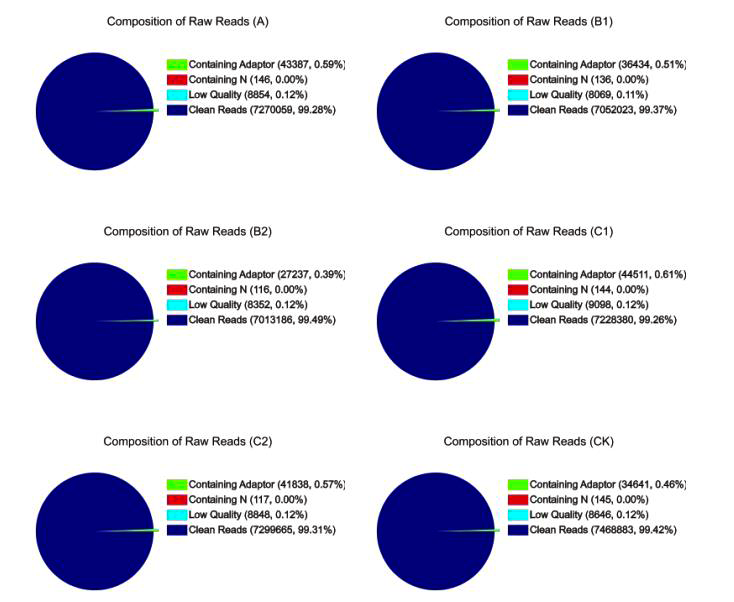

Supplement: Supplementary file 1 — Additional file 1: Figure S1: Composition of raw reads in the six RNA libraries. “Clean” reads refers to those remaining after the removal of adaptor sequences, reads in which the proportion of missing bases was >10% and reads in which low quality (≤5) bases represented >50% of the reads. The numbers in parentheses indicate the percentage of each type of read present. (TIFF 616 KB) [file 12864_2014_6537_MOESM1_ESM.tiff]

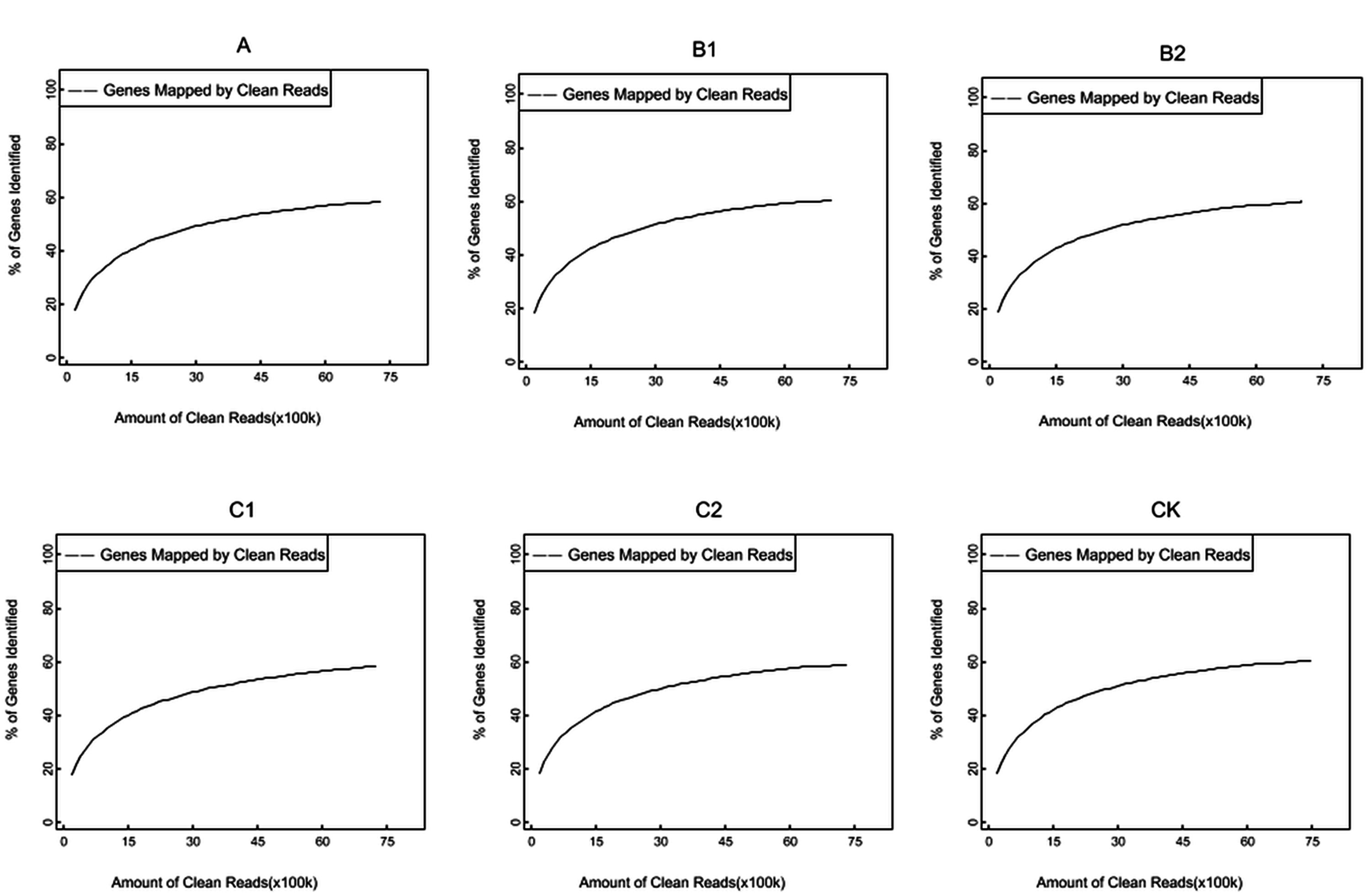

Supplement: Supplementary file 2 — Additional file 2: Figure S2: Sequencing saturation analysis in the six libraries (A, B1, B2, C1, C2 and CK). The numbers of new genes detected rose as the read number was increased, but not beyond a threshold around 7,000,000. (TIFF 797 KB) [file 12864_2014_6537_MOESM2_ESM.tiff]

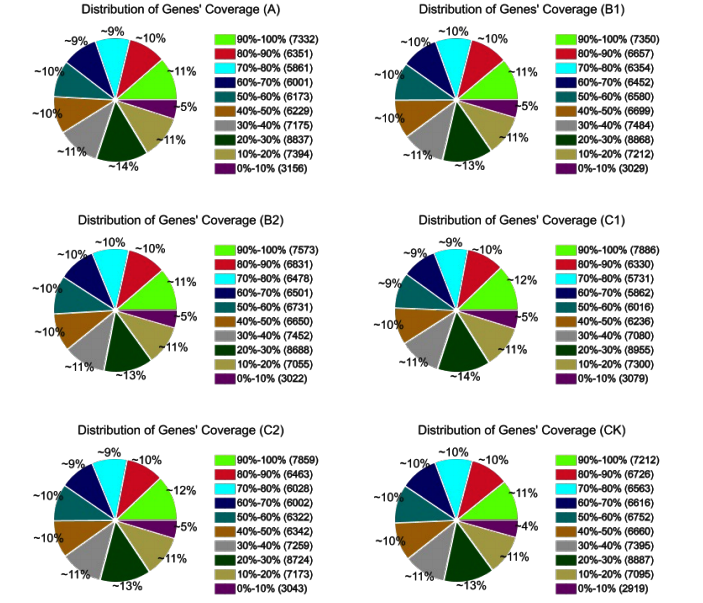

Supplement: Supplementary file 3 — Additional file 3: Figure S3: Distribution of gene coverage in the six libraries. (TIFF 783 KB) [file 12864_2014_6537_MOESM3_ESM.tiff]
